# Comprehensive Proteomic Profiling of Aldehyde Dehydrogenases in Lung Adenocarcinoma Cell Lines

**DOI:** 10.1155/2011/145010

**Published:** 2011-10-29

**Authors:** Qing Zhang, Ayumu Taguchi, Mark Schliekelman, Chee-Hong Wong, Alice Chin, Rork Kuick, David E. Misek, Samir Hanash

**Affiliations:** ^1^Molecular Diagnostics Program, Fred Hutchinson Cancer Research Center, 1100 Fairview Avenue North, M5-C800, Seattle, WA 98109, USA; ^2^University of Michigan Comprehensive Cancer Center, 8D17 300 NIB, University of Michigan, Ann Arbor, MI 48109, USA; ^3^Department of Surgery, University of Michigan, 1150 West Medical Center Drive, Room A510A MSRB-1, Ann Arbor, MI 48109, USA

## Abstract

We have explored the potential of proteomic profiling to contribute to the delineation of the range of expression and subcellular localization of aldehyde dehydrogenases (ALDHs) in lung adenocarcinoma. In-depth quantitative proteomics was applied to 40 lung adenocarcinoma cell lines resulting in the identification of the known members of the ALDH family. Substantial heterogeneity in the level and occurrence of ALDHs in total lysates and on the cell surface and in their release into the culture media was observed based on mass spectrometry counts. A distinct pattern of expression of ALDHs was observed in cells exhibiting epithelial features relative to cells exhibiting mesenchymal features. Strikingly elevated levels of ALDH1A1 were observed in two cell lines. We also report on the occurrence of an immune response to ALDH1A1 in lung cancer.

## 1. Introduction

Aldehyde dehydrogenases (ALDHs) constitute a large family of enzymes that catalyze the oxidation of endogenous and exogenous aldehydes to carboxylic acids. The different family members exhibit heterogeneous tissue distribution and have been localized predominantly in the cytosol and mitochondria. ALDHs have been investigated primarily based on their gene expression, their immunohistochemical localization and based on their activity. Cell sorting techniques have been utilized to enrich for cells expressing these enzymes based on activity. The relevance of ALDHs to cancer stems in part from the role they may play during carcinogenesis, their association with therapeutic resistance, and more recently from a distinct pattern of expression of ALDH1A1 and ALDH3A1 in cancer stem cells, which has been exploited as a means to define this cell population in tumors [1–3]. Several studies have pointed to evidence for epithelial to mesenchymal transitions in cancer stem cells, defined, in part, based on their ALDH expression or activity [4, 5]. While ALDH1 and 3 dominate the cancer stem cell literature, other ALDHs have also been explored. High-level expression of ALDH1B1 was observed in colon cancer by immunohistochemistry [6]. Moderate to strong staining for ALDH4A1, ALDH5A1, and ALDH6A1 was observed in most cancer tissue in Protein Atlas (http://www.proteinatlas.org/). Strong expression of ALDH7A1 has been found in human prostate cancer cell lines, primary tumors, and matched bone metastases, with evidence of its functional involvement in the formation of bone metastases [7]. Comparative analysis of hepatocellular carcinoma tissue and adjacent nontumor tissue identified changes in ALDHs1-3 proteins in tumor tissue [8]. 

Several studies have explored the biological significance of ALDHs specifically in lung cancer and have provided supportive evidence for the association between ALDH activity and lung cancer stem cells [9]. Flow cytometric analysis of a panel of lung cancer cell lines and patient-derived tumors revealed the occurrence of a subpopulation of cells with elevated ALDH activity in most non-small cell lung cancers, which correlated with ALDH1A1 expression [10]. Immunohistochemical staining of a large panel of primary tumors revealed a significant correlation between ALDH1A1 expression and poor prognosis in patients, including those with early stage disease [10]. Likewise, in another study, expression of ALDH1 was found to be positively correlated with the stage and grade of lung tumors and related to a poor prognosis for patients with early-stage lung cancer [11]. Expression analysis of sorted cells revealed elevated Notch pathway transcript expression in ALDH-positive cells. Suppression of the Notch pathway resulted in a significant decrease in ALDH-positive lung cancer cells with concordant reduction in tumor cell proliferation and clonogenicity [10]. Downregulation of ALDH isozymes affects cell growth, cell motility, and gene expression in lung cancer cells [12]. Other ALDHs have also been explored in lung cancer. ALDH3B1 is expressed in a tissue-specific manner and in a limited number of cell types. ALDH3B1 expression was found to be upregulated in a high percentage of human tumors, particularly lung tumors [13].

In general, most studies of ALDHs in lung cancer have focused on particular members of the family. In-depth proteomic profiling allows delineation of proteins expressed in tumor cells and in subcellular compartments. In this study we applied quantitative in-depth proteomic profiling to assess the occurrence of ALDHs in whole lysates of 40 lung adenocarcinoma cell lines and to examine their association with the cell surface and the extent of their release into culture media. ALDH1A1 was further explored as a tumor antigen that induces an autoantibody response in lung cancer.

## 2. Methods 

### 2.1. Lung Adenocarcinoma Cell Line Culture

Cells were grown in DMEM media (Invitrogen) containing 0.1% of dialyzed fetal bovine serum (FBS) (Invitrogen) and ^13^C-lysine instead of regular lysine, for 7 passages (1 : 2) according to the standard SILAC protocol [14]. Incorporation of ^13^C Lys isotope exceeded 90% of the total protein lysine content. The same batch of cells was used for extracting cell surface proteins and for analysis of conditioned media and whole-cell lysate proteins. The secreted proteins were obtained directly from the cell conditioned media after 48 h of culture. Cells and debris were removed by centrifugation at 5000 ×g and filtration through a 0.22 *μ*M filter. Total extracts of cells were obtained by sonication of ~2 × 10^7^ cells in 1 mL of PBS containing the detergent octyl glucoside (OG) (1% w/v) and protease inhibitors (complete protease inhibitor cocktail, Roche Diagnostics, Germany) followed by centrifugation at 20,000 ×g. 

### 2.2. Capture of Cell Surface Proteins

To isolate cell surface proteins, ~2 × 10^8^ cells were biotinylated in the culture plate after extensive PBS rinsing, with 10 mL of 0.25 mg/mL of Sulfo-NHS-SS-BIOTIN in PBS at room temperature (23-24°C) for 10 min. The residual biotinylation reagent was quenched with 10 mM lysine. Protein extraction was performed in a solution containing NP 40 detergent 2% (v/v) with cell disruption by sonication followed by centrifugation at 20,000 ×g. Biotinylated proteins were chromatographically isolated by affinity chromatography using 1 mL of UltraLink Immobilized NeutrAvidin (Pierce) according to manufacturers' instruction. Proteins bound to the column were recovered by reduction of the biotinylation reagent with 5 mL of a solution containing 65 *μ*Mol of DTT and 1% octyl glucoside (OG) detergent for 1 h at 37°C. Eluted proteins were subsequently alkylated with 200 *μ*Mol of iodoacetamide at room temperature.

### 2.3. Fractionation of Cell Extracts

Cell surface, conditioned media, and total extract were fractionated by reversed-phase chromatography, using, respectively 500 *μ*g, 1 mg, and 1 mg of total protein. All the cell extracts were reduced and alkylated with iodoacetamide prior to chromatography. Separation was performed in a POROS R1/10 column (Applied Biosystems—4.6 × 50 mm) at 2.7 mL/min using a linear gradient of 10 to 80% of organic solvent over 30-minute run. Solvent system used was aqueous solvent, 5% acetonitrile/95% water/0.1% of trifluoroacetic acid; organic solvent, 75% acetonitrile/15% isopropanol/10% water/0.095% trifluoroacetic acid. Fractions were collected at a rate of 3 fractions/minute.

### 2.4. Protein Identification by LC-MS/MS

Protein digestion and identification by LC-MS/MS was performed as described previously [15]. Briefly, each one of the reversed-phase fractions was individually digested in-solution digestion with trypsin (400 ng/fraction) and grouped into 24 to 27 pools for each cell line and each compartment (i.e., cell surface, conditioned media, and soluble whole-cell lysate) based on chromatographic features. Pools were individually analyzed by LC-MS/MS in a LTQ-Orbitrap mass spectrometer (Thermo-Finnigan) coupled to a nanoflow chromatography system (Eksigent) using a 25 cm column (PicoFrit 75 *μ*M ID, New Objectives, packed in-house with Magic C18 resin) over a 90-minute linear gradient. Acquired data was automatically processed by the Computational Proteomics Analysis System (CPAS) [16]. The tandem mass spectra were searched against version 3.57 of the human IPI database. A variable modification of 6.020129 mass units was added to lysine residues for database searching to account for incorporation of the heavy lysine isotope. We applied the tools PeptideProphet [17] and ProteinProphet [18] to estimate the significance of peptide and protein matches. Identifications with a PeptideProphet probability of greater than 0.2 were selected and submitted to ProteinProphet. The latter infers a minimal set of proteins that explain the peptide evidence, assigning a probability to each protein based on the combined peptide probabilities. The derived protein identifications were filtered at a <5% error rate based on the probability that the best match obtained would fall in the distribution of random database matches [19]. A spectral counting method [20] was used to estimate protein enrichment for each compartment. 

### 2.5. Two-Dimensional Polyacrylamide Gel Electrophoresis (2D PAGE)

Proteins derived from the extracts of the cultured H522 lung adenocarcinoma cell line were separated into two dimensions as described previously [21]. Briefly, cultured NCI-H522 cells were lysed in solubilization buffer (8 M urea (Bio-Rad), 2% Nonidet P-40, 2% carrier ampholytes, pH 4–8 (Gallard/Schlesinger, Carle Place, NY), 2% ß-mercaptoethanol, and 10 mM PMSF). 200 *μ*g of solubilized proteins were applied onto isoelectric focusing gels. Isoelectric focusing was performed using pH 4–8 carrier ampholytes at 700 V for 16 h, followed by 1000 V for an additional 2 h. The first-dimension gel was loaded onto the second-dimension gel, after equilibration in 125 mM Tris (pH 6.8), 10% glycerol, 2% SDS, 1% DTT, and bromophenol blue. For the second-dimension separation, a gradient of 11–14% acrylamide (Crescent Chemical, Hauppauge, NY) was used. The resolved proteins were transferred to an Immobilon-P polyvinylidene difluoride (PVDF) membrane (Millipore, Bedford, Mass). Protein patterns in some gels were visualized directly by silver staining or Sypro Ruby staining.

### 2.6. Western Blotting and Image Analysis

After transfer, membranes were incubated with a blocking buffer consisting of PBS and 0.1% Tween-20 containing 1.8% nonfat dry milk for 2 h. The membranes were incubated for 1 h at room temperature with serum obtained from either patients or control individuals as a source of primary antibody at a 1 : 100 dilution. Some additional membranes were incubated with an antibody to ALDH1A1 to visualize the protein. After three washes with washing buffer (PBS containing 0.1% Tween 20), the membranes were incubated with horseradish peroxidase-conjugated sheep anti-human IgG (Amersham Biosciences, Piscataway, NJ) at a dilution of 1 : 1000 for 1 h at room temp. Immunodetection was accomplished by enhanced chemiluminescence (Amersham Biosciences) followed by exposure on Hyperfilm MP (Amersham Biosciences). Films were digitized with a Kodak CCD camera. The spots on each image were detected and quantified as previously described [21]. After immunoblotting, all membranes were Coomassie Blue stained, and the patterns obtained were compared to those of the films in order to determine the locations of reactive spots. Spot integrated intensities were normalized by dividing by the intensity of a reference HSP27 protein spot. HRP-conjugated goat anti-mouse secondary antibody was spiked into the sheep anti-human cocktail at a 1 : 1000 dilution to facilitate detection.

### 2.7. Statistical Analysis

The data was analyzed using one-sided Rank-Sum tests.

## 3. Results

### 3.1. Occurrence of ALDHs among Lung Adenocarcinoma Cell Lines and in Subcompartments

Comprehensive proteomic profiling of 40 lung cancer cell lines resulted in the identification of known members of the ALDHs family in one or more cell lines. There was substantial heterogeneity in the abundance of individual family members among cell lines and in their occurrence and distribution between whole-cell lysates, on the cell surface and their release into the media of cultured cells (Table 1). ALDH1A1, ALDH1A3, ALDH2, ALDH3A1, ALDH7A1, and ALDH9A1 had more than 1,000 total MS counts each in total cell extracts across cell lines. ALDH1A1 and ALDH3A1 exhibited discordant expression with an overall total of MS counts for ALDH1A1 exceeding that of ALDH3A1 (Table 1), with some cell lines yielding high MS counts for ALDH1A1 with low counts or undetectable for ALDH3A1. The highest MS counts were observed for ALDH2 and ALDH1A1. However, whereas ALDH2 was relatively uniformly distributed across cell lines, expression of ALDH1A1 was more limited to just a few cell lines. Notably, cell line H522 yielded extremely high MS counts for ALDH1A1 relative to most other cell lines, and relative to other ALDHs (Table 2). Several ALDH proteins for which we provide evidence of expression in lung cancer cell lines (Table 1) were previously not characterized in lung cancer. ALDH9A1 was identified in most cell lines and was among the most abundant ALDH proteins identified based on spectral counts. 

### 3.2. Occurrence of ALDHs in the Extracellular Compartment

**Figure 1:**
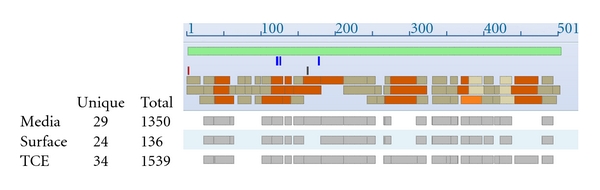
Extent of peptide coverage of ALDH1A1 in cell line H522 in the TCE, media, and cell surface, across the ALDH1A1 protein sequence. Blue bar: dbSNP; red bar: N-acetylserine; black bar: conflict, V->I at 162; tan bar: non-Cys-containing tryptic peptide; orange bar: Cys-containing tryptic peptide; light tan bar: N-linked non-Cys-containing tryptic peptide; light orange bar: N-linked Cys-containing peptide.

We sought to determine the occurrence of ALDHs on the cell surface and their release into culture media. Whereas all ALDH proteins were identified in total cell extracts (TCE), for most proteins a significant fraction was observed in both the cell surface fraction (Surf) and the media (Med) (Table 1). For example, ALDH1A1 in H522 was predominant in both the TCE and Med (Table 2) whereas other proteins (e.g., ALDH3A2 and ALDH5A1) displayed a substantial proportion of protein abundance on the cell surface (Table 1). We examined whether MS data was suggestive of the occurrence of cleavage forms of ALDHs in the media or on the cell surface. However, the data obtained pertaining to peptide representation of the proteins was not suggestive of cleavage, or alternatively spliced forms of proteins associated with the cell surface or, for that matter, with their release into the media as exemplified for ALDH1A1 in H522 (Figure 1).

### 3.3. Differential Expression of ALDHs Based on Epithelial/Mesenchymal Features

**Figure 2:**
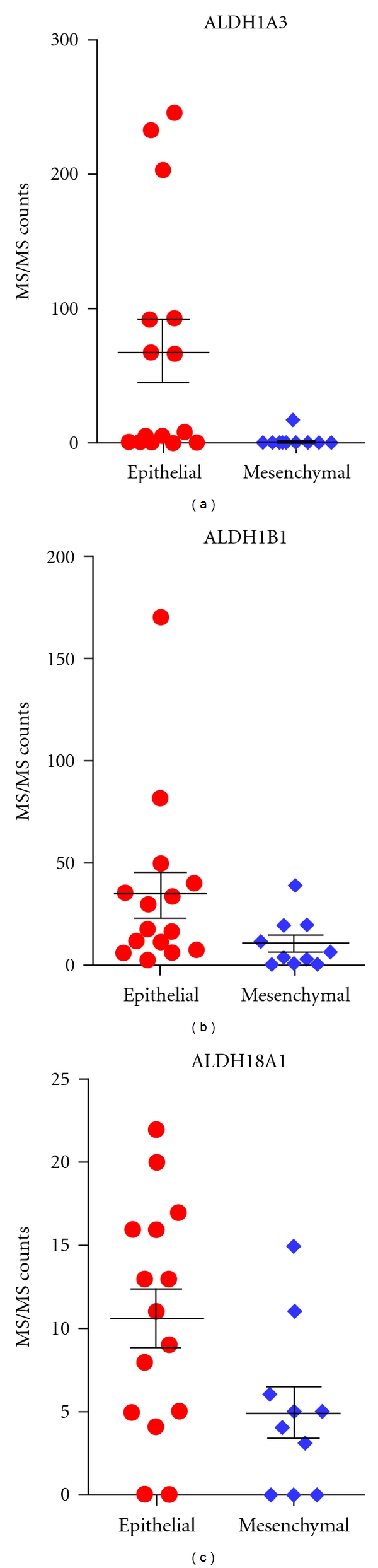
Differential expression of ALDH1A3, ALDH1B1, and ALDH18A1 between cell lines with epithelial and cell lines with mesenchymal features based on MS/MS counts. A Mann-Whitney test was performed using MS/MS counts of epithelial and mesenchymal cell lines. ALDH1A3 and ALDH1B1 have significantly higher expression from TCE in epithelial cells as compared to mesenchymal ones, with a *P* value of 0.007 and 0.030, respectively. ALDH18A1 has significant higher expression from the cell surface in epithelial as compared to mesenchymal with a *P* value of 0.042.

**Figure 3:**
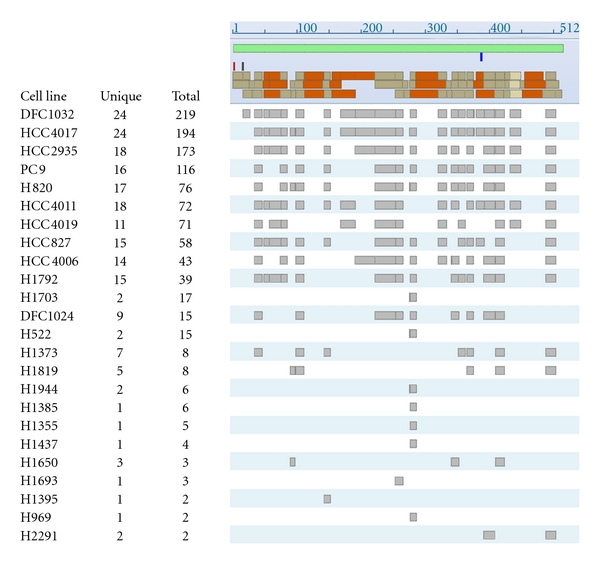
Extent of peptide coverage of ALDH1A3 across cell lines based on spectral counts from MS data.

Twenty-five of the 40 cell lines investigated could be classified as either epithelial or mesenchymal based on their morphology and their expression of vimentin and E-cadherin. ALDH protein expression was examined in relation to cell line epithelial/mesenchymal characteristics. ALDH1A3, ALDH1B1, and ALDH18A1 exhibited statistically significant differences in their MS counts in epithelial versus mesenchymal cell lines (Table 3, Figure 2). Whereas the stem cell markers ALDH1A1 and ALDH3A1, did not yield statistically significant differences between epithelial and mesenchymal cell lines, a very high number of MS spectral counts were observed for ALDH1A1 in the two mesenchymal cell lines H522 and H1703 (Table 3). The extent of peptide coverage for ALDH1A3, ALDH1B1, and ALDH18A1 between epithelial and mesenchymal cell lines appears to be related to the total number of MS counts rather than to epithelial/mesenchymal characteristics, as shown in Figure 3 for ALDH1A3 in TCE. 

### 3.4. Lung Cancer Sera Exhibit IgG-Based Reactivity to ALDH1A1

**Figure 4:**
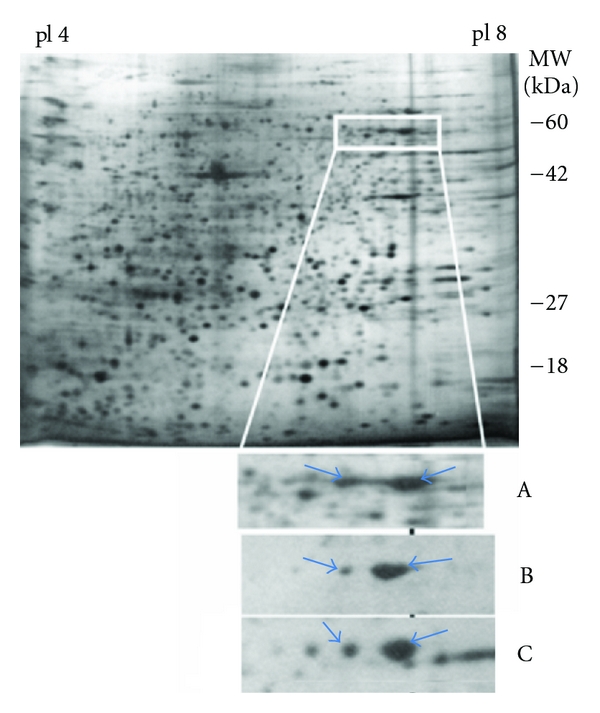
Occurrence of autoantibodies to ALDH1A1 in lung-cancer. A rectangle marks the location of ALDH1A1 in a silver-stained 2D gel of H522 lysate, also shown in a close-up below (A). The acidic and basic ALDH1A1 reactive spots in Western blots hybridized with subject sera (B and C) are indicated with arrows.

H522 tumor cell line proteins were separated by 2D PAGE and transferred onto Immobilon-P PVDF membranes, and the membranes were used to screen individual lung cancer and control sera for autoantibodies directed against H522 proteins. Sera from 25 lung cancer patients (9 adenocarcinomas, 6 small cell, and 10 squamous lung cancers) and 25 age- and sex-matched healthy controls were investigated. Two neighboring spots, both with similar apparent molecular weight (approximately 55 kDa) but slightly different isoelectric points (6.6 and 6.45, resp.), were observed to exhibit frequent reactivity in lung cancer patients (Figure 4). The data for the more acidic spot was highly correlated to the more basic spot values, giving a Spearman's rank correlation of 0.62 with a *P* value of 2.3 × 10^−6^. The more basic spot was excised from gels on two occasions and digested with trypsin, with the resulting tryptic digests subjected to mass spectrometric analysis. We obtained 46 and 45 spectra matching the aldehyde dehydrogenase 1A1 protein (NP_000680, gene symbol ALDH1A1) on the two runs. These matches amounted to 26 and 22 distinct peptides, with MASCOT search engine scores of 1503 and 1464, respectively. The more acidic spot was also identified as ALDH1A1, with 21 matching peptides and MASCOT score of 1329.

Spot integrated intensities on the films were measured and analyzed by one-sided Rank-Sum tests. We obtained estimated *P* values of 0.0018 (basic) and 0.0026 (acidic) from 10000 permuted data sets, indicating greater reactivity in lung cancer patients compared to healthy controls. Two receiver operating characteristic (ROC) curves were constructed, each using the data for a single protein spot (either the more basic or more acidic spot). For the more basic spot, an AUC of 0.69 was obtained. An AUC of 0.68 was obtained for the more acidic spot. 

## 4. Discussion 

Comprehensive proteomic profiling delineated the range of expression and localization of ALDHs in total cell lysates, the cell surface, and their release into the media of cultured lung adenocarcinoma cell lines. A striking finding is the extent to which ALDHs localize to the cell surface and/or are released into the media. The evidence obtained based on the peptides identified for various ALDHs does not suggest the occurrence of distinct forms of ALDHs in different compartments, thus the mechanisms involved and the role of ALDHs localized to the cell surface or release into the media largely remain to be determined.

We observed a distinct pattern of expression of ALDHs in cell lines exhibiting epithelial versus mesenchymal features. In other studies, ALDH expression was found to mark pancreatic cancer cells that have stem cell and mesenchymal features [22]. ALDH expression was analyzed by immunohistochemistry in 269 primary surgical specimens of pancreatic adenocarcinoma and examined for association with clinical outcomes and in paired primary tumors and metastatic lesions from eight pancreatic cancer patients who had participated in a rapid autopsy program. The clonogenic growth potential of ALDH-positive pancreatic adenocarcinoma cells was assessed in vitro by a colony formation assay and by tumor growth in immunodeficient mice. ALDH-positive tumor cells were detected in 90 of the 269 primary surgical specimens, and their presence was associated with worse survival. Six of the eight patients with matched primary and metastatic tumor samples had ALDH-negative primary tumors, and in four of these six patients, the matched metastatic lesions contained ALDH-positive cells which expressed genes consistent with a mesenchymal state [22]. 

The extent to which mesenchymal features correlate with a stem cell phenotype reflected in a common ALDH expression pattern remains to be determined. In our studies, ALDH1A1 and ALDH3A1, which have been associated with stem cell features, exhibited distinct expression patterns among the cell lines analyzed. ALDH3A1 did not yield a significant association with a mesenchymal phenotype whereas ALDH1A1 exhibited strikingly high expression based on MS counts in two cells lines with a mesenchymal phenotype. Moreover, ALDH1A3, ALDH1B1, and ALDH18A1 exhibited a statistically significant difference in their protein expression between cell lines with epithelial and mesenchymal features.

We utilized lysates from H522, a lung adenocarcinoma cell line that expresses high levels of ALDH1A1 to characterize the humoral immune response in lung cancer. We obtained evidence for autoreactivity against two forms of ALDH1A in sera from subjects with lung cancer relative to healthy controls. The reactivity observed was not limited to lung adenocarcinoma, as sera from subjects with squamous cell lung cancer and small cell lung cancer exhibited similar reactivity. An immune response to ALDH1A1 has been reported in other studies. ALDH1A1 was identified as a novel CD8+ T-cell-defined tumor antigen in squamous cell carcinoma of the head and neck [23]. Mass spectral analysis of peptides in tumor-derived lysates was used to determine that the CTL line recognized the HLA-A2 binding ALDH1A1 (88–96) peptide. ALDH1A1(88–96) peptide-specific CD8(+) T cells recognized only HLA-A2(+) cell lines which over expressed ALDH1A1 and cells transfected with ALDH1A1 cDNA. In another study, sera from three of five patients with lung adenocarcinoma and none of ten controls with lung tuberculosis were found to exhibit IgG-based seroreactivity against aldehyde dehydrogenase identified in A549 lung adenocarcinoma cell line lysate using an approach similar to the approach for IgG reactivity against ALDH1A1 utilized in this study [24].

## Figures and Tables

**Table 1:** Identified Aldehyde Dehydrogenases in the total cell extracts (TCE), Media (Med) and the cell surface (Surf) across 40 lung adenocarcinoma cell lines based on mass spectrometry (MS/MS counts for corresponding peptides).

Gene symbol	Protein description	MS/MS counts (TCE)	MS/MS counts (Med)	MS/MS counts (Surf)	Unique peptides	Chromosome	Length	MW
ALDH16A1	Aldehyde dehydrogenase family 16 member A1	31	79	81	19	19	802	85127
ALDH18A1	Delta-1-pyrroline-5- carboxylate synthase	354	5	344	20	10	795	87302
ALDH1A1	Retinal dehydrogenase 1	4961	2640	1463	37	9	500	54731
ALDH1A2	Retinal dehydrogenase 2	10	1	1	5	15	480	53060
ALDH1A3	Aldehyde dehydrogenase family 1 member A3	1267	256	299	31	15	512	56108
ALDH1B1	Aldehyde dehydrogenase X, mitochondrial	843	22	384	29	9	517	57217
ALDH1L1	Aldehyde dehydrogenase family 1 member L1	5	3	3	8	3	505	55394
ALDH1L2	Aldehyde dehydrogenase family 1 member L2, mitochondrial	11	0	51	15	12	923	101776
ALDH2	Aldehyde dehydrogenase, mitochondrial	4964	889	1665	32	12	517	56381
ALDH3A1	Aldehyde dehydrogenase, dimeric NADP-preferring	1149	44	116	27	17	453	50379
ALDH3A2	Fatty aldehyde dehydrogenase	772	44	457	23	17	485	54848
ALDH3B1	Aldehyde dehydrogenase family 3 member B1	3	0	20	6	11	468	51840
ALDH3B2	Aldehyde dehydrogenase family 3 member B2	3	0	0	2	11	385	42670
ALDH4A1	Delta-1-pyrroline-5- carboxylate dehydrogenase, mitochondrial	256	0	41	22	1	563	61719
ALDH5A1	Succinate-semialdehyde dehydrogenase, mitochondrial	437	8	192	19	6	535	57215
ALDH6A1	Methylmalonate-Semialdehyde dehydrogenase [acylating], mitochondrial	131	0	13	17	14	535	5784
ALDH7A1	Alpha-aminoadipic semialdehyde dehydrogenase	2268	95	563	29	5	510	55235
ALDH8A1	Aldehyde dehydrogenase family 8 member A1	2	0	0	2	6	487	53401
ALDH9A1	4-trimethylaminobutyral dehyde dehydrogenase	1096	98	365	22	1	491	53374

**Table 2:** Identified Aldehyde Dehydrogenases in cell line H522.

Gene symbol	MS/MS counts (TCE)	MS/MS counts (Med)	MS/MS counts (Surf)
ALDH16A1	4	1	1
ALDH18A1	31	0	6
ALDH1A1	2053	1706	173
ALDH1A3	0	0	1
ALDH1B1	39	0	24
ALDH1L2	0	0	3
ALDH2	29	3	47
ALDH3A1	0	0	8
ALDH3A2	3	0	2
ALDH4A1	15	0	0
ALDH5A1	15	0	5
ALDH7A1	164	2	26
ALDH9A1	21	0	8

**Table 3:** Occurrence of ALDH1A1, ALDH3A1, ALDH1A3, ALDH1B1 and ALDH18A1 in cell lines with epithelial or mesenchymal features.

Cell Lines	EMT	ALDH1A1			ALDH3A1			ALDH1A3			ALDH1B1			ALDH18A1		
		TCE	Med	Surf	TCE	Med	Surf	TCE	Med	Surf	TCE	Med	Surf	TCE	Med	Surf
H1437	Epithelial	82	2	0	0	6	2	0	0	0	33	0	9	18	0	16
H1650	Epithelial	0	0	0	0	0	0	3	0	0	11	0	5	4	0	0
H3255	Epithelial	0	0	0	0	0	0	0	0	0	2	0	0	8	0	5
HCC4019	Epithelial	0	0	0	0	0	0	93	0	19	29	0	6	0	0	0
DFC1032	Epithelial	0	0	0	0	0	3	247	112	77	17	0	7	0	0	16
HCC827	Epithelial	0	0	0	0	0	2	65	0	0	81	1	14	14	0	5
H1573	Epithelial	0	0	0	129	0	0	0	0	0	6	0	0	1	0	13
HCC4011	Epithelial	0	0	0	0	0	0	91	10	5	40	0	12	12	0	13
H1819	Epithelial	0	0	0	0	0	0	8	0	0	6	0	3	6	0	9
H1395	Epithelial	8	0	0	4	3	8	0	0	0	49	0	12	9	0	4
H969	Epithelial	134	108	33	7	1	0	0	0	0	11	0	6	62	0	20
H820	Epithelial	0	0	0	0	0	4	67	2	14	171	14	66	27	3	17
H2291	Epithelial	0	0	0	0	0	0	3	0	2	7	0	19	8	2	22
HCC4017	Epithelial	0	0	0	0	0	0	234	93	5	35	1	3	9	0	8
HCC2935	Epithelial	5	0	0	0	6	0	205	8	73	16	0	3	2	0	11

H1299	Mesenchymal	0	0	0	4	0	0	0	0	2	3	0	5	0	0	3
H23	Mesenchymal	0	0	0	0	0	0	0	0	0	0	0	13	0	0	0
H838	Mesenchymal	0	0	0	0	0	1	0	0	2	0	0	2	0	0	0
H2030	Mesenchymal	0	0	0	0	0	0	0	0	0	0	0	0	0	0	5
H650	Mesenchymal	0	0	0	0	0	0	0	0	0	6	0	4	0	0	0
DFC1024	Mesenchymal	0	0	0	0	0	0	17	0	42	20	0	6	2	0	5
H1355	Mesenchymal	34	2	0	0	0	13	0	0	1	11	0	7	17	0	11
H2405	Mesenchymal	2	0	0	0	0	41	0	0	0	2	0	9	5	0	4
H522	Mesenchymal	2053	1706	173	0	0	8	0	0	1	39	0	24	31	0	6
H1703	Mesenchymal	1665	354	78	0	0	7	0	0	18	20	1	30	10	0	15
